# Acute pulmonary embolism in a patient with hepatitis C virus infection and hepatocellular carcinoma: a case report

**DOI:** 10.1186/s43044-021-00193-2

**Published:** 2021-07-31

**Authors:** Mahmoud Abdelnabi, Yehia Saleh, Abdallah Almaghraby, Özge Özden Tok, Hoda Abdelgawad, Sherif Abd ElSamad

**Affiliations:** 1 Cardiology and Angiology Unit, Department of Clinical and Experimental Internal Medicine, Medical Research Institute, Alexandria University, Alexandria, Egypt; 2 Cardiology Department, Houston Methodist Hospital, Houston, TX USA; 3 Cardiology Department, Faculty of Medicine, University of Alexandria, Alexandria, Egypt; 4 Cardiology Department, Memorial Bahçelievler Hospital, Istanbul, Turkey

**Keywords:** Hepatitis C virus, Pulmonary embolism, Hepatocellular carcinoma, Echocardiography

## Abstract

**Background:**

Cardiac metastases in hepatocellular carcinoma patients are infrequently encountered and usually associated with a very poor prognosis.

**Case presentation:**

Hereby, we report a case of an acute pulmonary embolism (PE) on top of HCC with direct cardiac invasion to the right atrium (RA) through the inferior vena cava with another metastasis to the right ventricular apex in the form of highly mobile cauliflower mass protruding through the tricuspid valve into RA and nearly obliterating right ventricular outflow tract in a multi-centric hepatocellular carcinoma patient.

**Conclusion:**

Acute dyspnea in a patient with a long history of hepatitis C virus infection raises the suspicion of acute PE due to either hypercoagulable state induced by malignancy or by cardiac extension of the tumor which usually carries high mortality rates. To the best of our knowledge, this case is the first case in the literature to show cardiac metastases in HCC with two different pathological mechanisms.

**Supplementary Information:**

The online version contains supplementary material available at 10.1186/s43044-021-00193-2.

## Background

Cardiac complications of hepatocellular carcinoma (HCC) are quite rare. Right atrial invasion with right ventricular outflow obstruction and Budd Chiari syndrome was previously reported while cardiac metastases in HCC patients are rarely encountered and mostly are associated with high mortality.

## Case presentation

**Fig. 1:**
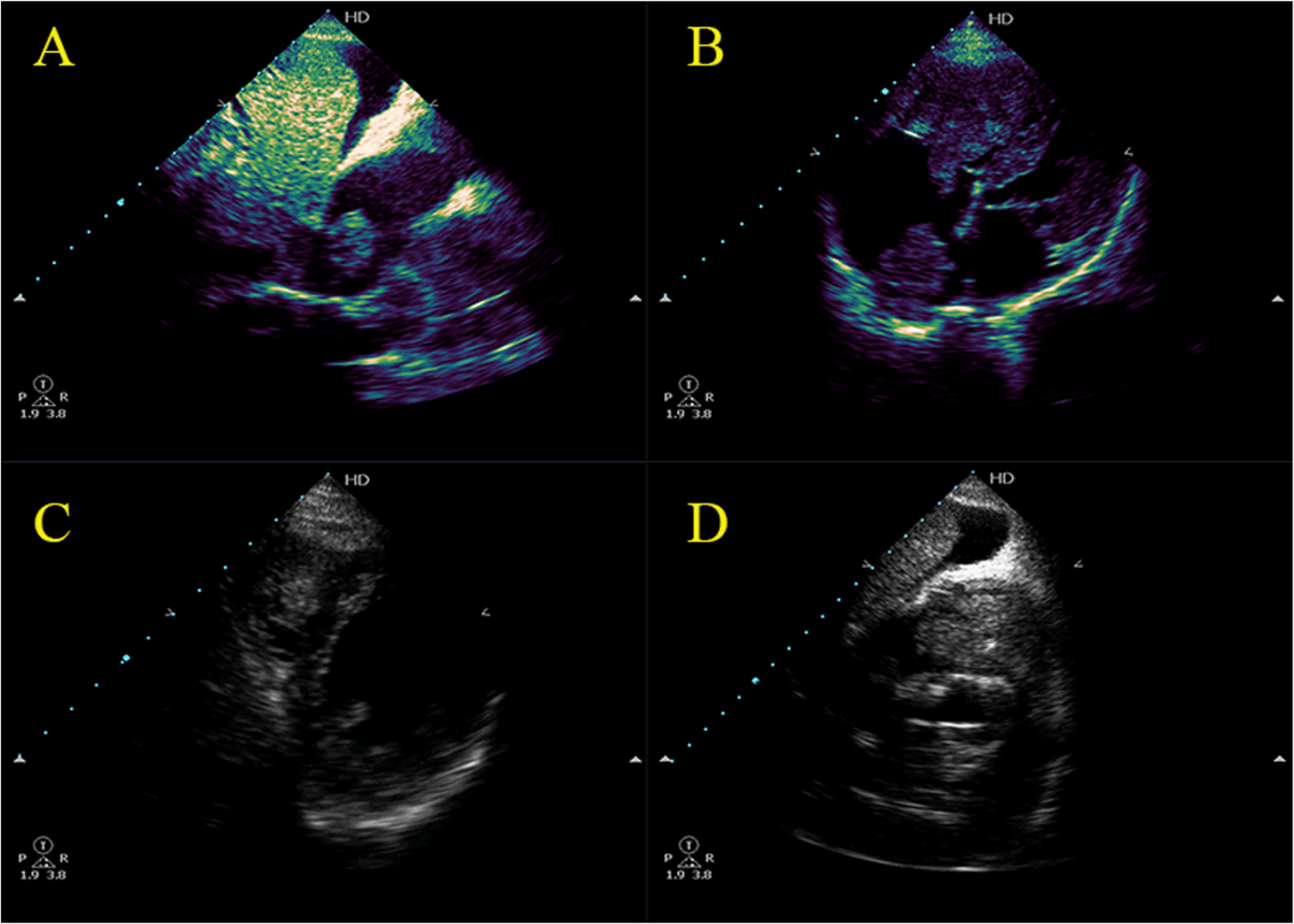
**a**–**d** Transthoracic Echocardiography revealing a large solid mass extending through IVC to RA with another highly mobile cauliflower mass at the RV apex occupying The RV cavity, protruding into RA through TV and nearly obliterating RVOT into the pulmonary artery

A 72-year-old male patient with a history of hepatitis C virus (HCV) for 20 years yet did not receive any treatment. He was admitted to our medical facility for resolved hepatic encephalopathy, decompensated liver failure, and acute renal failure due to hepatorenal syndrome type I. Suddenly while admitted, he started to complain of acute onset of acute chest pain, severe dyspnea, and tachycardia. On clinical examination, he was distressed with a deep icteric tinge, tachypneic with a thready pulse, massive ascites, and bilateral lower limb pitting edema. His vital signs showed a heart rate of 120 bpm, blood pressure of 90/60, temperature of 36.5 °C, respiratory rate of 30, O_2_ saturation of 92% on 2 L nasal cannula. There was a loud S2 on cardiac auscultation, but the rest of his physical exam was unremarkable. Electrocardiogram (ECG) revealed sinus tachycardia and s1q3t3 pattern. Urgent transthoracic echocardiography (TTE) was done revealing a large solid mass extending through the inferior vena cava (IVC) to the right atrium (RA) with another highly mobile cauliflower mass at the right ventricular (RV) apex occupying the RV cavity, protruding into RA through TV and nearly obliterating RV outflow tract into the pulmonary artery (Fig.1, video 1). Ultrasound abdomen confirmed the presence of multi-centric hepatocellular carcinoma (HCC) with direct invasion to the IVC therefore, he was diagnosed with acute pulmonary embolism (PE) due to tumor thrombus metastasis of HCC to the heart. Due to patient frailty, hazards of dye in an already renally impaired patient after patient and cardiothoracic surgery counseling, no further computed tomography pulmonary angiography (CTPA) or triphasic CT of the abdomen were done. Only conservative supportive measures were initiated to stabilize the deteriorated general condition but regretfully, he passed away shortly after diagnosis.

## Discussion

Involvement of the heart in HCC is rarely encountered and usually develops in advanced stages of HCC [1]. Previously reported cardiopulmonary complications of HCC included right-sided heart failure, tricuspid stenosis or insufficiency, RVOT obstruction, acute PE, and sometimes sudden cardiac death [2]. Direct tumor thrombus extension through hepatic veins and IVC is the main mechanism of metastasis to the heart [1]. Acute pulmonary embolism in the setting of HCC is a far infrequent manifestation of HCC that usually occurs due to tumor thrombi in the IVC, RA, and R V [3]. Cardiac surgery and urgent thrombectomy have been proposed in selected patients with a satisfactory general condition [3]. Unfortunately, intracardiac involvement in HCC carries a very poor prognosis with a mean survival of 1 to 4 months at the time of diagnosis [2].

To the best of our knowledge, this case is the first case in the literature to demonstrate cardiac metastases to several cardiac chambers, to RA by direct extension through IVC as well as the RV mostly by hematogenous spread.

## Conclusion

Acute dyspnea in a patient with HCC raises the suspicion of acute PE induced by either a hypercoagulable state in malignancy or by tumor thrombus through malignant cardiac extension.

## Supplementary Information

**Additional file 1: Video 1.** Transthoracic Echocardiography revealing a large solid mass extending through IVC to RA with another highly mobile cauliflower mass at the RV apex occupying The RV cavity, protruding into RA through TV and nearly obliterating RVOT into the pulmonary artery.

## Data Availability

The data is available for sharing

## Abbreviations

HCC: Hepatocellular carcinoma
PE: Pulmonary embolism
RA: Right atrium
HCV: Hepatitis C virus
ECG: Electrocardiogram
TTE: Transthoracic echocardiography
IVC: Inferior vena cava
RV: Right ventricle
CTPA: Computed tomography pulmonary angiography

## References

[1] Abdelnabi M, Almaghraby A, Saleh Y, Abd ES (2019). Hepatocellular carcinoma with a direct right atrial extension in an HCV patient previously treated with direct-acting antiviral therapy: a case report. Egypt Heart J.

[2] Sung AD, Cheng S, Moslehi J, Scully EP, Prior JM, Loscalzo J (2008). Hepatocellular carcinoma with intracavitary cardiac involvement: a case report and review of the literature. Am J Cardiol.

[3] Lin H-H, Hsieh C-B, Chu H-C, Chang W-K, Chao Y-C, Hsieh T-Y (2007). Acute pulmonary embolism as the first manifestation of hepatocellular carcinoma complicated with tumor thrombi in the inferior vena cava: surgery or not?. Dig Dis Sci.

